# The Terneuzen Birth Cohort: BMI Changes between 2 and 6 Years Correlate Strongest with Adult Overweight

**DOI:** 10.1371/journal.pone.0009155

**Published:** 2010-02-11

**Authors:** Marlou L. A. De Kroon, Carry M. Renders, Jacobus P. Van Wouwe, Stef Van Buuren, Remy A. Hirasing

**Affiliations:** 1 Department of Public and Occupational Health, The EMGO Institute for Health and Care Research, VU University Medical Centre, Amsterdam, The Netherlands; 2 Netherlands Organisation for Applied Scientific Research, TNO Quality of Life, Prevention and Health Care, Leiden, The Netherlands; 3 Department of Methodology and Statistics, Faculty of Social Sciences, University of Utrecht, Utrecht, The Netherlands; UCL Institute of Child Health, United Kingdom

## Abstract

**Background:**

Complications of overweight amplify with age, and irreversible damage already exists in young persons. Identifying the most sensitive age interval(s) for adult overweight is relevant for primary prevention. The aim of the study was to assess the relative contribution of body mass index (BMI) changes between 0 and 18 years to adult overweight, and to identify the earliest critical growth period.

**Methods and Findings:**

Data from 762 subjects in the Terneuzen Birth Cohort with an average of 21 growth measurements per subject from birth until 18 years were used. The main outcome measure was the BMI standard deviation score (SDS) at young adulthood. For each subject BMI SDS was fitted by a piecewise linear model at eight different ages and correlated to adult BMI SDS. The age intervals in between are considered critical according to three criteria, tested by respectively Students' *t*-tests, multiple linear regression analyses and Pearson's correlation tests. In the age intervals 4 months(m) -1 year(y), 2–6 y, 6–10 y and 10–18 y the BMI SDS change differs between adults with and without overweight (*P*≤0.001). The age intervals 2–6 y and 10–18 y also meet the second criterion, implying that the BMI change during this period has a predictive value for adult BMI SDS in addition to BMI SDS at the end of the period. The largest rise in correlation between estimated BMI SDS and measured adult BMI SDS occurs during the period 2–6 y (from 0.36 to 0.63), which results in a high sensitivity (0.6) and specificity (0.8) by the age of 6 y.

**Conclusions/Significance:**

The age interval from 2 y to 6 y is the earliest and most critical growth period for adult overweight. Therefore, primary prevention of adult overweight seems most likely to be successful if targeted at this specific age interval. By identifying those with an upwards centile crossing between 2 and 6 years, the development towards adult overweight might be reversed.

## Introduction

The effect of overweight on later cardiovascular health problems amplifies with age [1], and irreversible precursors of diabetes and cardiovascular disease already exist in young persons [2]. Not only is weight in itself a risk factor, but so is also a fast BMI increase during childhood [1], [3]–[7]. For the prevention of adult overweight, research has focused on the identification of sensitive or so-called ‘critical’ growth periods. A growth period is critical for adult overweight if changes within this period increase the risk of adult overweight [8]. Several investigators have distinguished growth periods with increased risk [4], [9]–[17].

**Figure 1:**
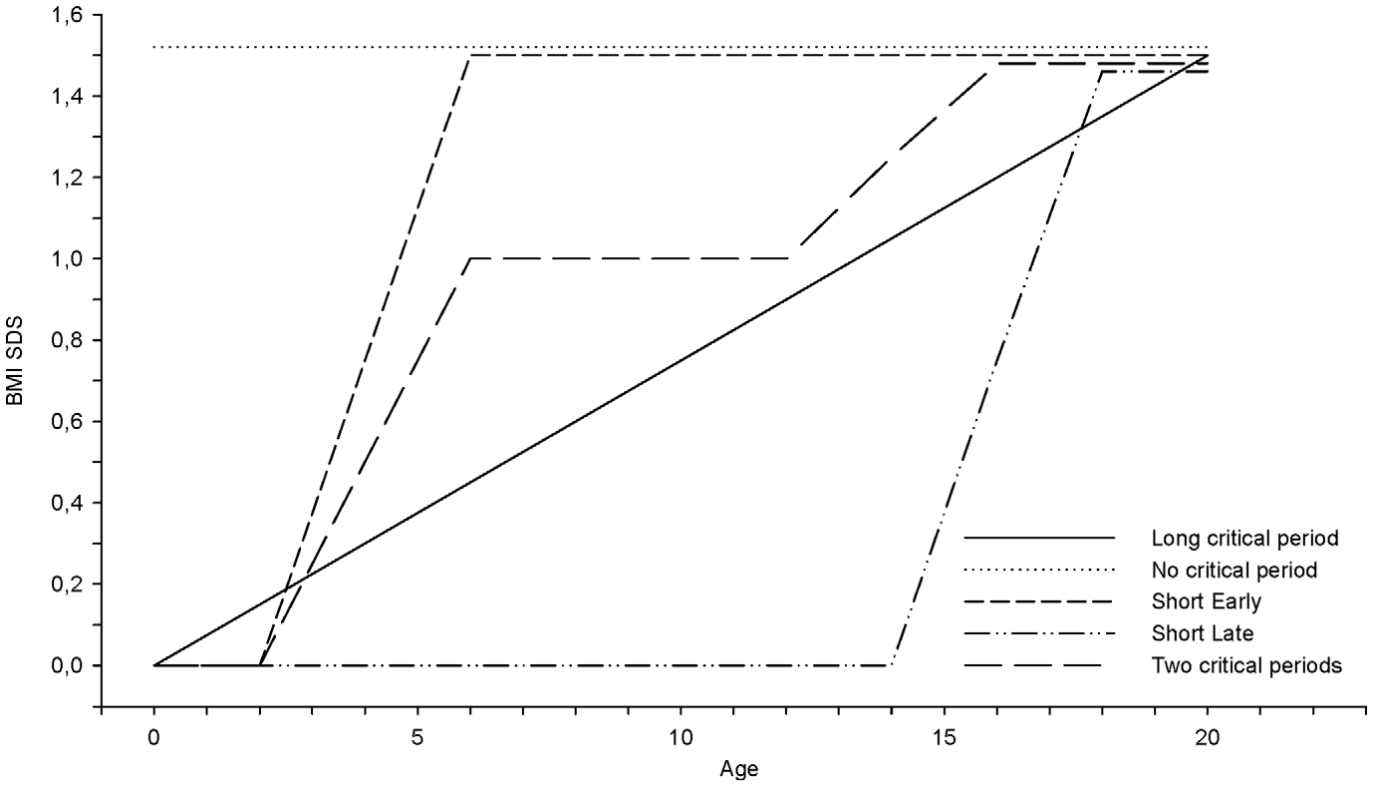
Five hypothetical trajectories towards overweight.

Figure 1 illustrates how the BMI standard deviation score (BMI SDS) in five hypothetical growth patterns evolves into adult overweight. The first pattern is a simple trajectory with a constant increase in BMI SDS over a prolonged time interval, e.g. 0–20y. Every period seems to be critical here (a ‘long critical’ period). Another simple trajectory occurs if children are already overweight at birth and remain overweight until adulthood, so in essence no critical period exists (‘no critical’ period). By contrast, the ‘short early’ and ‘short late’ trajectories have large increases in BMI SDS during short time periods. The rise in BMI SDS could also be broken into a smaller number of critical periods, e.g. ‘two critical’ periods. The last three patterns (‘short early’, ‘short late’ and ‘two critical’ periods) suggest that prevention opportunities are to be found before or within the periods of BMI SDS increase, rather than after. In all situations statistical evidence is required to confirm that changes in BMI SDS effectively influence the risk of adult overweight.

Few studies have followed the BMI changes in children from birth to adulthood; most studies limit themselves to a time interval during childhood [9], [10], [12]–[14], [18]–[21] or have a follow-up that does not exceed puberty [11], [12], [17], [20], [21]. Also, their results are sometimes contradictory [6], [16], [20], [21]. Two recent studies without these shortcomings included respectively males only [16] and no Caucasians [15].

We aim to assess the relative contribution of BMI SDS changes between 0–18 y of age to adult overweight, and to identify the earliest relevant, critical growth period for adult overweight.

## Methods

### Ethics Statement

The study protocol was approved by the Medical Ethics Committee of the VU University Medical Centre Amsterdam, and written informed consent was obtained from all participants.

### Population and Study Design

The original cohort consists of all 2,604 children born between 1977 and 1986 in the city of Terneuzen. Of the 1,701 subjects data for weight and length as routinely registered by the Municipal Health Services were available from birth. Of these subjects, 762 persons (45%) were willing to participate in a follow-up study at young adulthood that included measurements of weight, height and waist circumference and a questionnaire to collect sociodemographic characteristics. This is described in more detail elsewhere [22]. The participants in the follow-up study did not differ from the original cohort regarding baseline characteristics collected at birth, e.g. date of birth, birth weight, BMI SDS at birth, age of the mother, and parity, except for gender (41% were males vs 51% in the original cohort, *P*<0.05). We used BMI values (kg/m^2^) as the measure for (over-)weight, converted to age-specific standard deviation scores (BMI SDS) based on Dutch reference data [23], because these are most comparable to our study population. The criterion for being overweight in young adulthood is defined as BMI SDS≥1.3 (roughly a BMI≥25).

In contrast to most studies that are limited to a specific period (infancy, childhood or adolescence) and lack of follow-up to adulthood, our cohort covers the complete growth from birth to adulthood. For comparison purposes with other studies, we divided the growth period of our cohort into the following age intervals: 0–8 days (0–8 d) [9], 8 days-4 months (8 d-4 m) [9], 4 months-1 year (4 m-1 y)[24], 1-2 years (1–2 y) [4], 2–6 years (2–6 y) [12], 6–10 years (6–10 y), and 10–18 years (10–18 y) [14], [25]. The upper limit in the age interval 6–10 y was set since Dutch children go into puberty after 10 years of age; the upper limit of 18 years marks the start of adulthood. The limits of all periods (0 d, 8 d, 4 m, 1 y, 2 y, 6 y, 10 y and 18 y) are called *break ages*.

### Statistical Analysis

The major analytic problem was that the number and the timing of the measurements vary between individuals. We solved this by fitting each individual BMI SDS trajectory by a piecewise linear model, otherwise known as a broken stick-model [26], with the knots set equal to the break ages. We also dealt with missing data in this way. This model approximates each person's observed BMI SDS trajectory by a series of straight lines that connect to each other at the break ages. In order to stabilize the parameter estimates, we fitted these parameters as randomly varying slopes in a linear multilevel model [27]. We used the S Plus 8.0 function bs() to code the data into the appropriate form, and used the function lme() to estimate the parameters as random effects. The procedure resulted in eight parameters per person that together describe the persons' BMI SDS trajectory. Each parameter corresponds to the predicted value for each individual, using both random and fixed estimates. We call these *status scores*. They are represented as Z_0d_, Z_8d_, and so on. The change in BMI SDS per period is equivalent to the difference between two successive *status scores*, i.e. Z_8d_-Z_0d_, Z_4m_-Z_8d_, and so on. We call these *change scores*.

We define a growth period, bounded by ages T1 and T2, as critical if:

- the mean *change score Z*_T2_-*Z*_T1_ is significantly different between those with and without adult overweight,
- the *change score Z*_T2_-*Z*_T1_ and *Z*_T2_ are both significantly related to adult BMI SDS in a multiple regression analysis, which is, as has been suggested by Lucas [28], equivalent to the significance of *Z*_T1_ as predictor in addition to the significance of *Z*_T2_ as predictor (see Addendum S1 for further explanation), and
- the score *Z*_T2_ is relatively close to BMI SDS at adult age.

Criterion *a* will filter out periods during which the two mean curves of the BMI SDS trajectory diverge, so significant differences in growth of those who do and those who do not become overweight emerge. Criterion *b* indicates if the preceding *change score* has additional value to the *status score* at the end of the period, in predicting the BMI SDS at adulthood. Criterion *c* will select periods for which it is easier (i.e. with higher sensitivity and specificity) to identify children at risk for adult overweight.

We tested for these criteria in SPSS 14.0 by applying Student's *t*-tests (2-sided), Pearson's correlation coefficients and multiple regression analysis (with alpha = 0.05 for statistical significance). In the multiple regression analyses multiplicative interaction effects were entered to explore whether early weight is modifying the effect of later weight size on adult overweight with a type I error rate of 0.10 [28]. Age, gender, parity, exclusive breastfeeding (<90 vs ≥90 days) were included to study potential confounding or effect modification.

## Results

The mean age of the 762 subjects is 23.1 (SD 2.9). No difference in baseline characteristics between males (n = 307) and females (n = 455) were found (*P*>0.05). An average number of 21 growth measurements per participant between 0 y and 18 y were performed. Table 1 provides baseline characteristics at birth and adulthood and the average number of growth measurements per age interval.

**Table 1:** General characteristics at birth and at adulthood, number of subjects (N), and their mean (SD) number of height and weight measurements per age interval.

	**Males**				**Females**			
**Characteristics**	***N***	***Mean***	***SD***		***N***	***Mean***	***SD***	
birth weight (g)	830	3481.4	549.3		870	3348.2	541.0	
birth length (cm)	804	50.9	2.5		839	50.2	2.2	
gestational age (wk)	765	39.9	1.7		819	39.8	2.4	
adult height (cm)	307	182.6	6.8		455	169.6	6.2	
adult weight (kg)	307	77.0	12.3		455	67.8	11.9	
adult BMI (kg/m^2^)	307	23.1	3.4		455	23.4	3.9	
***measurements:***	***height***		***weight***		***height***		***weight***	
**Age interval**	*N*	*mean (SD)*	*N*	*mean (SD)*	*N*	*mean (SD)*	*N*	*mean (SD)*
0-8 d	810	1.0 (0.1)	1311	2.6 (1.8)	852	1.0 (0.1)	1284	2.5 (1.9)
8 d-4 m	735	2.8 (1.1)	754	4.3 (1.7)	799	2.7 (1.1)	818	4.5 (1.9)
4 m-1 y	751	4.8 (1.6)	753	5.4 (1.8)	815	4.8 (1.5)	818	5.6 (2.0)
1–2 y	709	1.8 (0.7)	710	1.8 (0.8)	765	1.8 (0.7)	767	1.8 (0.7)
2–6 y	802	2.7 (0.9)	804	2.7 (1.0)	848	2.7 (0.9)	850	2.7 (0.9)
6–10 y	734	1.6 (0.6)	735	1.6 (0.6)	787	1.6 (0.7)	788	1.6 (0.7)
10–18 y	723	1.7 (0.8)	724	1.7 (0.8)	766	1.6 (0.7)	767	1.6 (0.7)

**Figure 2:**
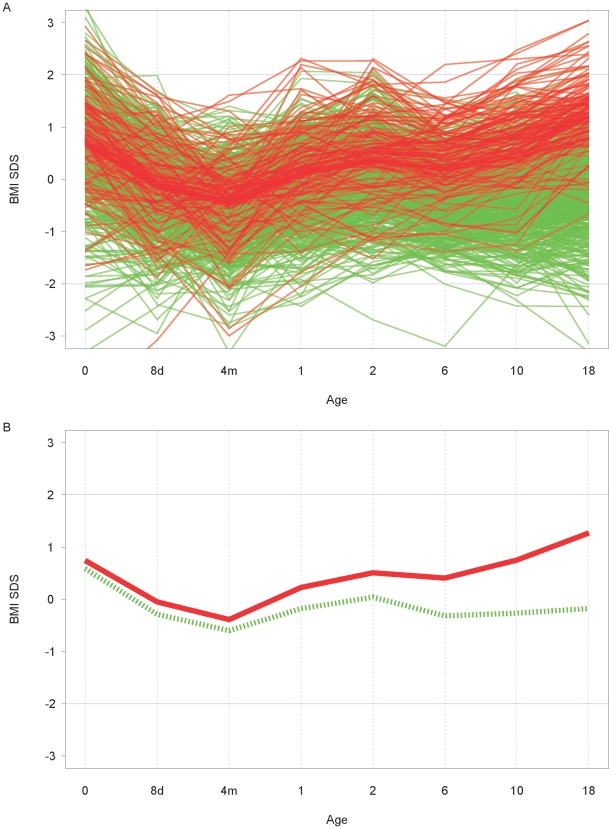
Broken sticks trajectories of BMI SDS changes. A. Broken sticks trajectories for subjects with normal weight (green lines) versus subjects with overweight (red lines) at young adulthood, B. Broken-stick model of mean increments for subjects with normal weight and with overweight at young adulthood. The green, dotted line represents the mean increments of subjects with normal weight, the red line the mean increments of subjects with overweight at young adulthood.

Figure 2a shows the fitted broken sticks trajectories for each subject. Figure 2b demonstrates the means of the broken sticks trajectories for young adults with normal weight and overweight. It is noteworthy that those with overweight track differently: the *change scores* differ, i.e. the lines are not parallel with those of normal weight.

We tested criterion *a* by Student's *t*-tests applied to the *change scores* at successive age intervals. Based on the recommendations of Jones and Spiegelhalter [29], we applied the analyses to unconditional *change scores*, because the correlations between subsequent values of the BMI SDS at the break-ages were substantially higher than 0.5, except for a slightly lower correlation between the BMI SDS at 8 days and 4 months (ρ = 0.48). Significant differences were found for four age intervals: 4 m-1 y, 2–6 y, 6–10 y and 10–18 y. No differences in the *change scores* were found for the age interval 1–2 y (Table 2). Similar results were found for males and females, although for the age interval 4 m-1 y the difference became non-significant in males (*P* = 0.078). In addition *t*-tests were applied to test differences in BMI SDS changes for those with or without increased waist circumference at young adulthood as defined by IDF criteria. These analyses showed significant results for exactly the same intervals (*P*<0.001).

**Table 2:** Mean *change score* (SE) per age interval for subjects with adult normal weight (BMI SDS <1.3, n = 608) and with overweight (BMI SDS *≥*1.3, n = 154).

Age interval	Mean *change score* (SE) for adults with normal weight	Mean *change score* (SE) for adults with overweight	*P* (*t*-test)
0–8 d	−0.87 (0.024)	−0.79 (0.055)	0.145
8 d-4 m	−0.31 (0.030)	−0.34 (0.065)	0.703
4 m-1 y	0.42 (0.025)	0.62 (0.056)	0.001
1–2 y	0.22 (0.020)	0.28 (0.044)	0.183
2–6 y	−0.36 (0.018)	−0.10 (0.041)	<0.001
6–10 y	0.05 (0.014)	0.35 (0.029)	<0.001
10–18 y	0.09 (0.027)	0.42 (0.043)	<0.001

The results of the multiple regression analyses to test criterion *b* are shown in Table 3, in which adult BMI SDS is the outcome, and the *status score(s)* the predictor(s). Because no effect-modification was found for gender (*P*>0.3), in applying multiple regression analyses and correlation coefficients males and females could be analyzed as one group, increasing statistical power. As parity and breastfeeding duration did not influence the results (*P*>0.05), these variables were not included in the final models. Not surprisingly, in the simple linear regression analyses BMI SDS is significantly related to adult weight at all ages. After including the previous *status score* as a second (linear) predictor, only two age intervals, 2–6 y and 10–18 y, met both criterion *a* and criterion *b*. Moreover, these periods are both characterized by significant predictors with opposite regression signs, which means that especially the BMI SDS changes in these age intervals are relevant [28]. We extended the smaller time intervals between birth and the age of 2 years to one age interval, in order to assess if the length of the age intervals influenced the results of the analyses. However, no significant effect has been shown by adding the *status score* at birth to the *status score* at 2 y: the increase in explained variance is zero; (β in the multiple regression model at 2 y and at 0 d are respectively 0.548 (SE 0.06, *P*<0.001) and 0.039 (SE 0.046, *P* = 0.394).

**Table 3:** Linear relation between BMI SDS at young adulthood and BMI SDS at earlier age: Model A includes one *status score* as independent variable (a), model B is model A extended with the preceding BMI SDS^#^ (b) as independent variable.

Independent variables	Models A*				Models B*			
	*β* &	*SE*	*P*	*Adj R^2^*	*β_1_* &	*SE*	*P*	*Adj R^2^*
					*β_2_* &			
(a) BMI sds at birth	0.158	0.047	0.001	0.035	–	–	–	–
–					–	–	–	
(a) BMI sds at 8 d	0.320	0.057	<0.001	0.060	0.390	0.084	<0.001	0.061
(b) BMI sds at birth					−0.077	0.069	0.260	
(a) BMI sds at 4 m	0.307	0.060	<0.001	0.054	0.210	0.066	0.002	0.071**
(b) BMI sds at 8 d					0.239	0.063	<0.001	
(a) BMI sds at 1 y	0.562	0.057	<0.001	0.138	0.591	0.068	<0.001	0.138
(b) BMI sds at 4 m					−0.052	0.071	0.464	
(a) BMI sds at 2 y	0.559	0.053	<0.001	0.146	0.346	0.081	<0.001	0.158**
(b) BMI sds at 1 y					0.291	0.083	0.001	
(a) BMI sds at 6 y	1.095	0.049	<0.001	0.407	1.583	0.077	<0.001	0.454**
(b) BMI sds at 2 y					−0.557	0.069	<0.001	
(a) BMI sds at 10 y	1.014	0.035	<0.001	0.530	1.126	0.080	<0.001	0.530
(b) BMI sds at 6 y					−0.155	0.099	0.177	
(a) BMI sds at 18 y	1.065	0.020	<0.001	0.790	1.292	0.041	<0.001	0.790**
(b) BMI sds at 10 y					−0.305	0.047	<0.001	

* Intercepts not reported ^#^Interaction effects between BMI SDS at the end and at the start of the periods were not included in models B, because they were all non-significant,

** F-test for comparing the multiple regression model with the simple regression model is significant (*P*<0.001),

& values of β, β_1_ and β_2_ are adjusted for gender and the age at the measurement of BMI SDS at young adulthood, – Not applicable, *Adj R^2^* adjusted variance.

The increase of explained variance caused by including BMI-SDS at T0 into the model containing BMI-SDS at T1 was largest for the period 2–6 years. This implicates that the influence of the *change scores* on adult overweight is largest for the age interval 2–6 y. Because the relative changes in regression signs after extending the models is highest in the age interval 2–6 y, especially in this age interval upwards centile crossing is an additional risk to the *status score* at the end of these age intervals (see Addendum S1). For comparisons reasons with a recent study [16], we added an additonal breakpoint at 4 y, and found that the proportion of increased variance as a function of the *status score* at the end of the period for the age intervals 2–4 y and 4–6 y are respectively 0.05 and 0.04 by adding the *status score* at the start of the period to the model. In modeling the Z-score of the waist circumference at young adulthood as the outcome measure (number of missing outcomes is 5), we obtained similar results for the age interval 2–6 y. In the multiple regression the coefficients of the *status scores* at 6 y and at 2 y coefficients are respectively 0.31 (SE 0.09, *P*<0.001) and −0.14 (SE 0.08, *P* = 0.048), with an increased explained variance of 3% by augmenting the model with the preceding BMI SDS. Finally, because extreme high BMI at adulthood is more closely related to fat mass than lower values of BMI, we performed additional analyses by using adult obesity (BMI**≥**30) as the outcome. These analyses identified only the period 2–6 y as critical (OR of BMI SDS at respectively 6 y and 2 y were 41.27, 95%CI 15.8–107.7 and 0.24, 95%CI 0.12–0.50), whereas none of the other periods were found to comply with the conditions of a critical period.

**Figure 3:**
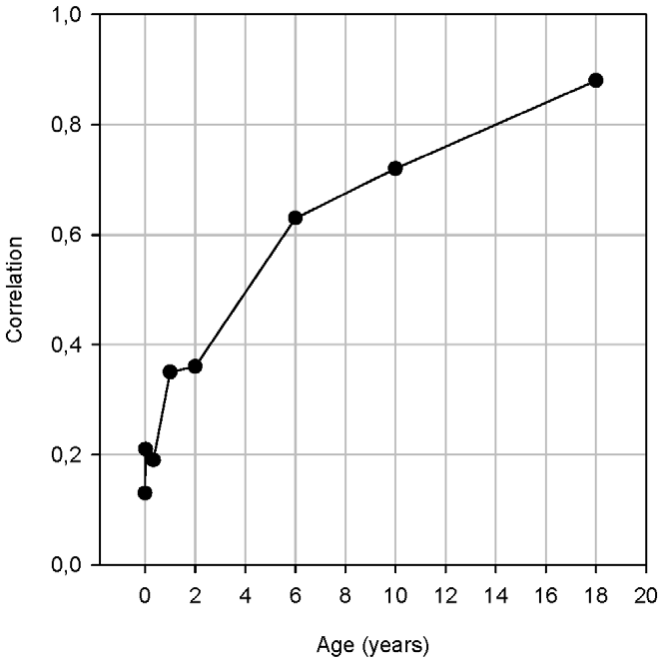
The correlation (Y-axis) of BMI SDS at several ages (X-axis) with the BMI SDS at young adulthood.

Criterion *c* was assessed by Pearson's correlation between *status scores* and adult BMI SDS (Figure 3). From the age of 6 years onwards the correlation between the *status score* and adult BMI SDS is greater than 0.6, which implies that prevention of a (relatively) high BMI SDS at the age of 6 y is relevant in terms of health outcome at adulthood.

Table 4 summarizes previous results by age interval. It appears that the age intervals 2–6 y and 10–18 y fulfill all criteria for the definition of a critical growth period for adult overweight. The age interval 2–6 y is the earliest growth period fulfilling these criteria.

**Table 4:** Summary of the results of the analyses and the interpretation per age interval based on criteria a, b and c.

Age interval	*Criterion a.* Relation with adult overweight according to Students *t* tests	*Criterion b.* Significance of Z_T1_ as predictor in addition to the significance of Z_T2_ as predictor in a multiple linear regression analyses	*Criterion c.* High correlation of BMI SDS at a certain age with BMI SDS at adulthood at the end of the period	Critical age interval based on results of analyses concerning *criteria a, b and c*.	Confirmation of other study results [reference(s)]
0–8 d	NS	NS	no	no	no [9]
8 d-4 m	NS	yes**	no	no	no [9], [10], [17]
4 m-1 y	yes*	NS	no	no	partly, concerning results of *t*-tests [10]
1–2 y	NS	yes*	no	no	yes [17]
2–6 y	yes**	yes**	yes**	yes	yes [5], [15], [21]
6–10 y	yes**	NS	yes**	yo	possibly, not validated yet [16]
10–18 y	yes**	yes**	yes**	yes	yes [25]

NS not statistically significant (p>0.05),

*P = 0.001,

**P<0.001.

## Discussion

This paper addresses the issue whether sensitive or so-called critical periods in human growth exist during which BMI SDS changes have a significant impact on adult overweight. Our study results show that the *change score* during the age interval between 2 and 6 years is the earliest period with an effect on adult overweight. Moreover, the effect of this period is more substantial than the effects we found for other periods. This result indicates that the age interval 2–6 years is especially important to develop strategies for primary prevention of overweight. Our study shows that two children with identical BMI SDS at age 6 y have different risks for becoming overweight depending on their BMI SDS at 2 y. Also the correlation with adult overweight rises most during this age period (from 0.36 to 0.63), indicating that the rise in sensitivity and specificity for predicting adult overweight based on childhood BMI SDS in this period is high. Ideally, primary prevention should be realized before the point of high sensitivity and specificity has been reached. For the age interval 10–18 y a similar relation between *change score* and adult overweight is found, although weaker. In contrast during the age interval 6–10 y and up to the age of 2 years, *change scores* are not very predictive for adult overweight.

At first sight, our results deviate from the GOOD study in young male adults [16]. In this study both early and late childhood (defined as 1–4 y and 4–10 y) were found to be predictors of adult BMI. Their breakpoint was chosen at 4 y which is exactly in the middle of the age interval 2–6 y. By additional analyses, we found that the age intervals 2–4 y and 4–6 y are quite similar in terms of their predictive ability. Therefore it might be possible that the predictive value of the early child period in the GOOD study might be mainly explained by the predictive value of the period 2–4 years and the predictive value of the late childhood period mainly by the period 4–6 years.

We found that changes in BMI SDS up to the age of 2 years have hardly any predictive value for adult overweight. The *change score* in the period 4m-1y differs significantly between adults with and without overweight, but this effect disappears once BMI SDS at 1 y is included in the statistical model. Thus, a *change score* in the age interval from 4 months to 1 year of age seems not to correlate with a higher adult overweight risk at the age of 1 year.

Our study confirms the results from other studies that growth during certain age intervals in childhood are more sensitive in predicting overweight. However the explanation for these ‘critical’ growth periods is still unclear [4], [9], [12], [14], [24], [25]. It is possible that changing relations between BMI SDS and fat, lean and bone mass at different ages [30] and other biological explanations concerning the changing growth velocity of fat tissue play a role [15], [16], [17], [31], [32]. The results of this study did not show that rapid growth during the first years of life is a predictor for adult overweight, which is in contrast to the results from similar studies [9], [15], [17]. Possible explanations are a shorter follow-up [17], the selection of the study population [9], [15], or higher statistical power due to a larger study population [15]. Our conclusion that the age period between 2 and 6 years emerges to be critical for adult (over-)weight confirms other study results, that show a rapid elevation in the deposition of body fat rather than lean tissue mass just before the age of 6 years in children with a related early adiposity rebound (AR) [33], [34]. Other studies have also pointed to this crucial age period, with an early AR as a risk for adult overweight [4], [7], [9], [13], [20], [21]. The importance of adolescence for developing adult overweight was also reported in another study [16], which showed that changes in BMI SDS during adolescence reflect changes in visceral fat mass, more than in other periods.

The strenghts of our study are that it was carried out in a general population, and weight and height were frequently measured between 0 and 14 years according to the protocol used within Youth Health Care. The addition of protocolised measurements of weight and height at adulthood offered the opportunity to study the importance of all subsequent growth periods from birth to adulthood in the prediction of overweight at young adulthood. We also had to deal with limitations. As in most birth cohort studies, there was a substantial loss in the follow-up. Therefore sampling bias might be possible. However, there is no reason to assume that loss to follow up is related to the strength of the relation between BMI changes in childhood and adult BMI. Moreover, no significant differences were found for the baseline characteristics between the participants of the measurements and the other subjects of the original cohort except for gender.

Another limitation of our study is that we had to deal with missing data. This problem was solved by applying the broken stick method. The broken stick method results in estimates that are closer to the mean. This implies that any tests of differences will be conservative, and possibly underestimates the effects of BMI changes in periods in which fewer measurements are recorded. Also using BMI SDS (changes) as a predictor and as an outcome has limitations, although the correlation between BMI SDS and body fat% is reasonable and increases from 0.62 to 0.78 (between the ages of 3.5 and 7 years) [21]. Post hoc analyses with waist circumference, a proxy of central fat tissue considered most harmful to health [31], [35], and with adult obesity as the outcome measure, showed similar results for the period 2–6y. This, strengthens our impression that BMI SDS change, especially in the period 2–6 y, has a strong relationship with bodyfat% over the years. More fundamental research is needed to study the age dependency of the relation between BMI and several body components.

Our study indicates that the BMI change between 2 and 6 years of age (and, to a lesser degree, the age interval 10–18 y) has relatively the largest contribution to adult overweight. It would be interesting to study if in younger cohorts, living in an increasingly obesogenic society, the age interval between 2 and 6 years is also more predictive for adult overweight than other age intervals. If replicated in other studies, primary prevention of overweight should be more directed towards upwards centile crossing in the age interval 2–6 years. Especially in children with a normal weight, this may have a large payoff in terms of overweight reduction at adulthood, and the development towards adult overweight might be reversed.

## Supporting Information

Addendum S1 Explanation of criterion b of the definition of a critical growth period.
(0.02 MB DOC)
