# Supplementary material for: The Terneuzen Birth Cohort: BMI Changes between 2 and 6 Years Correlate Strongest with Adult Overweight
Source: PLoS One. 2010 Feb 11;5(2):e9155. doi: 10.1371/journal.pone.0009155 (PMC2820098; doi:10.1371/journal.pone.0009155)
Supplement: Addendum S1 — Explanation of criterion b of the definition of a critical growth period. (0.02 MB DOC) [file pone.0009155.s001.doc]

**Addendum a. Explanation of criterion *b* of the definition of a critical growth period**

As has been explained by Lucas et al[28], early size adjusted for later size is a measure of change of size (or centile crossing) between earlier and later measurement, especially if the regression coefficients have opposite signs. BMI SDS at adulthood (*Z*_A_) as outcome in a model with intercept α and the BMI SDS at the end of the period (*Z*_T2_) as well the BMI change (*Z*_T2_ - *Z*_T1_) during the period can be modeled by a regression model on *Z*_T2_ and *Z*_T1_ since

*Z*_A_ = α + β_4_ *Z*_T2_ + β_3_ (*Z*_T2_ - *Z*_T1_) + ε

= α + β_4_ *Z*_T2_  + β_3_ *Z*_T2_ – β_3_ *Z*_T1_ + ε

= α + β_2_ *Z*_T2_ + β_1_ *Z*_T1_  + ε

where β_2_ = β_3_+ β_4_ and β_1_ = - β_3_ generally will have opposite signs, which implicates that the change in BMI SDS during the period has additional predictive value, given for the BMI SDS at the end of the period.

In agreement with Lucas et al, we firstly entered age, gender and *Z*_T2_ to investigate the influence *Z*_T2_ on adult BMI SDS (model A of *Z*_T2_). Secondly, we replaced *Z*_T2_ by *Z*_T1_ to investigate the influence of *Z*_T1_ on adult BMI SDS (model A of *Z*_T1_). Then we entered both *Z*_T2_ and *Z*_T1_ to investigate if the change score (centile crossing) in the period T1-T2 is related to the BMI SDS at adulthood. Finally, we added the interaction term *Z*_T2_**Z*_T1_ to investigate if the value of *Z*_T1_  modifies the effect of *Z*_T2_ on outcome.
